# Draft Genome Sequences of *Klebsiella oxytoca* Isolates Originating from a Highly Contaminated Liquid Hand Soap Product

**DOI:** 10.1128/genomeA.00820-15

**Published:** 2015-07-23

**Authors:** J. A. Hammerl, P. Lasch, A. Nitsche, P. W. Dabrowski, H. Hahmann, A. Wicke, S. Kleta, S. Al Dahouk, R. Dieckmann

**Affiliations:** a Federal Institute for Risk Assessment, Department of Biological Safety, Berlin, Germany; b Robert Koch-Institut, ZBS 6, Proteomics and Spectroscopy, Berlin, Germany; c Robert Koch-Institut, ZBS 1, Highly Pathogenic Viruses, Berlin, Germany; d Landesamt für Verbraucherschutz Sachsen-Anhalt, Fachbereich Lebensmittelsicherheit, Halle, Germany

## Abstract

In 2013, contaminated liquid soap was detected by routine microbiological monitoring of consumer products through state health authorities. Because of its high load of *Klebsiella oxytoca*, the liquid soap was notified via the European Union Rapid Alert System for Dangerous Non-Food Products (EU-RAPEX) and recalled. Here, we present two draft genome sequences and a summary of their general features.

## GENOME ANNOUNCEMENT

Klebsiellae are nonmotile, Gram-negative, and opportunistic pathogens of the family *Enterobacteriaceae* and primarily infect immunocompromised patients during hospitalization. *Klebsiella pneumoniae* and *Klebsiella oxytoca* may cause pneumonia, antibiotic-associated hemorrhagic colitis, septicemia, and urinary tract and soft tissue infections (1). Hospital-acquired infections have been associated with inadequate disinfection (2) and with the use of hand sanitizer and liquid soap dispensers (3–5). Intrinsically and extrinsically contaminated personal care products have been notified as another source of infection (2, 4, 6–8).

Whole-genome sequencing was performed on *K. oxytoca* isolates recovered from two lots of liquid hand soap during official product quality testing in 2013. The bacteria were isolated using casein-peptone soymeal-peptone (CASO) agar (Merck, Darmstadt, Germany). Two passages of three independent batches were cultivated for 24 h at 37°C under aerobic conditions. Because of its high load of contamination (2.5 × 10^4^ CFU/g of soap), this soap was notified through the European Union Rapid Alert System for Dangerous Non-Food Products (EU-RAPEX) to be a microbiological risk and was therefore recalled (EU-RAPEX A12/1308/13). A deeper insight into the genomes of these *K. oxytoca* isolates may help to better understand how bacteria can survive in personal care products, how biocide tolerance mechanisms evolve, and whether cross-resistance to antimicrobials exists.

Cultivation of *K. oxytoca* strains was performed in LB broth for 24 h at 37°C under aerobic conditions. Isolation of genomic DNA (gDNA) was conducted using the QIAamp DNA minikit (Qiagen, Hilden, Germany), as recommended by the manufacturer. A total of 1 ng of gDNA was subjected to library preparation using the Illumina Nextera XT DNA sample preparation kit. Samples were tagged, pooled, and sequenced on a MiSeq with MiSeq paired-end (PE) 300 × 300-bp reads, using MiSeq reagent kit version 3 (Illumina, San Diego, CA). Genome assembly of strains PHS-890 and PHS-892 using Velvet (European Bioinformatics Institute) resulted in 86 and 85 contigs with sequence coverage of at least 80- and 30-fold per consensus base, respectively. BLAST genome comparison indicates that the closest relatives are *K. oxytoca* strains KONIH1 and M1 (9, 10). Genome annotation was performed with PGAP (http://www.ncbi.nlm.nih.gov/genome/annotation_prok) and revealed a similar genome composition of both strains, PHS-890 and PHS-892, consisting of 5,841 and 5,843 coding sequences (CDSs), 90 and 89 pseudogenes, and 22 and 25 rRNAs, respectively. Furthermore, 83 tRNAs, 10 noncoding RNAs (ncRNAs), 1 clustered regularly interspaced short palindrome repeat (CRISPR) array, and 11 putative prophage regions, of which four (phiP27, mEp460, PsP3, and SuMu) are intact, three (P4, P88, and HK225) are incomplete, and four (RE_2010, P88, phiES15, and ST64B) are questionable (11), were found on each genome. Bioinformatic analysis revealed that most of the predicted gene products are involved in metabolic activity, biogenesis, replication, recombination, and repair. Genome finishing may contribute to a better understanding of the influence of mobile genetic elements (e.g., plasmids and phages) on the adaption of *Klebsiella* to a stressful environment and the distribution of antibiotic resistance and/or biocide tolerance genes.

### Nucleotide sequence accession numbers.

The draft genome sequences (version 1) of the strains PHS-890 and PHS-892 have been deposited under GenBank accession numbers LDJV00000000 and LDJW00000000, respectively.
